# Dual roles of lactate and lactylation modification in the nervous system: neuroprotection and neuroinjury

**DOI:** 10.3389/fnagi.2026.1713583

**Published:** 2026-01-27

**Authors:** TianLu Ran, YunLong Shen, DeJian Peng, Li Tang, ZiHeng Pan, XinYi Zeng, Hui Liu

**Affiliations:** School of Basic Medicine, Yangtze University, Jingzhou, China

**Keywords:** lactate, lactylation modification, nervous system, neuroinjury, neuroprotection

## Abstract

Lactate is the terminal product of anaerobic oxidation within the glucose metabolism pathway. Traditionally, lactate has been regarded as a metabolically insignificant byproduct derived from incomplete oxidation. However, recent evidence suggests that lactate plays dual roles in the nervous system: neuroprotective and neurotoxicity. The diverse functions of lactate in the nervous system are influenced by its varying concentrations and distinct signal transduction pathways. This review focuses on elucidating the molecular mechanisms underlying lactate’s functions through energy metabolism, neurodegeneration, neural excitation, and neuroinflammation, particularly the signaling pathways involved in neuroprotection and neuroinjury. Furthermore, we highlight several pharmacological agents associated with these processes, aiming to provide novel insights and therapeutic strategies for neuroprotection under specific conditions such as hypoxia, and the management of neurological disorders.

## 1 Introduction

Lactate has traditionally been regarded as a waste product resulting from anaerobic oxidation of glucose. However, it has become evident that lactate serves a dual functions as both a metabolic energy source and a signaling molecule (Magistretti and Allaman, 2018), thus it exhibits the functions of both protection and injury. In neurophysiology, lactate exerts neuroprotective effects through multiple mechanisms, including energy metabolic reprogramming, activation of various signaling pathways, and protein lactylation (Chen et al., 2021). Nevertheless, under pathological conditions, the abnormal accumulation of lactate leads to cellular acidosis, mitochondrial dysfunction, and inflammatory cascades, which have emerged as critical drivers of neurodegenerative diseases (Li et al., 2022).

In cells, pyruvate is generated via glycolysis. In the presence of the coenzyme nicotinamide adenine dinucleotide (NADH), this process proceeds through the tricarboxylic acid (TCA) cycle to generate adenosine triphosphate (ATP), subsequently, ATP is produced through the TCA cycle (Rojas-Pirela et al., 2025). As an energy substrate, lactate is released into the bloodstream via monocarboxylate transporters (MCTs) and distributed throughout the body, serving as an efficient fuel source for vital organs such as the heart and brain (Hui et al., 2017; Rabinowitz and Enerbäck, 2020). Beyond its well-established role as an energy substrate, lactate serves as a biosynthetic precursor to promote the biosynthesis of sphingolipids, fatty acids, and amino acids (Chandel, 2021; de Kivit et al., 2024; Kuo and Hla, 2024).

Lactate acts as a signaling molecule primarily by binding to its specific receptor G Protein-Coupled Receptor 81 (GPR81, also known as HCAR1), playing an essential role in various physiological and pathological processes (Han Z. et al., 2025; He et al., 2025). On one hand, lactate binding to GPR81 activates the canonical Gi protein pathway, which ultimately suppresses lipolysis and modulates energy metabolism (Brooks, 2020; Yan C. et al., 2024; Lee et al., 2025). On the other hand, the lactate/GPR81 axis can also activate a non-canonical β-arrestin2-dependent route independently of Gi proteins (Yang et al., 2022), which consequently reduces the release of pro-inflammatory cytokines (Zhu et al., 2018; Gao et al., 2025) and suppresses Nuclear Factor-kappa B (NF-κB) activation, exerting broad anti-inflammatory effects (Wei et al., 2023; Wang Y. et al., 2024).

Lactate serves as a substrate for the lactylation of both histone and non-histone proteins, whereby a lactyl group is covalently attached to lysine residues (Chen et al., 2024; Li et al., 2024). Two acetyltransferases, p300 and HBO1, have been found to catalyze this process (Fan et al., 2023; Chen et al., 2024; Niu et al., 2024). Conversely, delactylase activity is attributed to Class I histone deacetylases (HDACs) and Certain Sirtuin Family Members (SIRTs), collectively modulating lactylation levels (Moreno-Yruela et al., 2022). Functionally, histone lactylation modulates gene transcription by directly altering chromatin structure, whereas non-histone lactylation regulates protein function, enzyme activity, and signaling pathways (Dong et al., 2024; Peng and Du, 2025; Sun C. et al., 2025).

Notably, lactate’s functions are environment-dependent dynamic response rather than static. This phenomenon is especially pronounced in the nervous system, which is highly complex both structurally and functionally. Although its systemic metabolic roles have been extensively studied, a systematic understanding of its concentration- and space-dependent effects in neural tissues remains limited. Unresolved questions encompass the concentration threshold of lactate, which governs its function switch from a “protective factor” to a “damaging factor,” and the molecular mechanisms underlying these functional transition in distinct neural cells. Against this backdrop, this review focuses on lactate’s roles in the nervous system, aiming to provide a theoretical framework for comprehending the lactate’s neurobiological functions and promoting related disease research.

## 2 Neuroprotective roles of lactate and lactylation modification

### 2.1 Energy support

**FIGURE 1:**
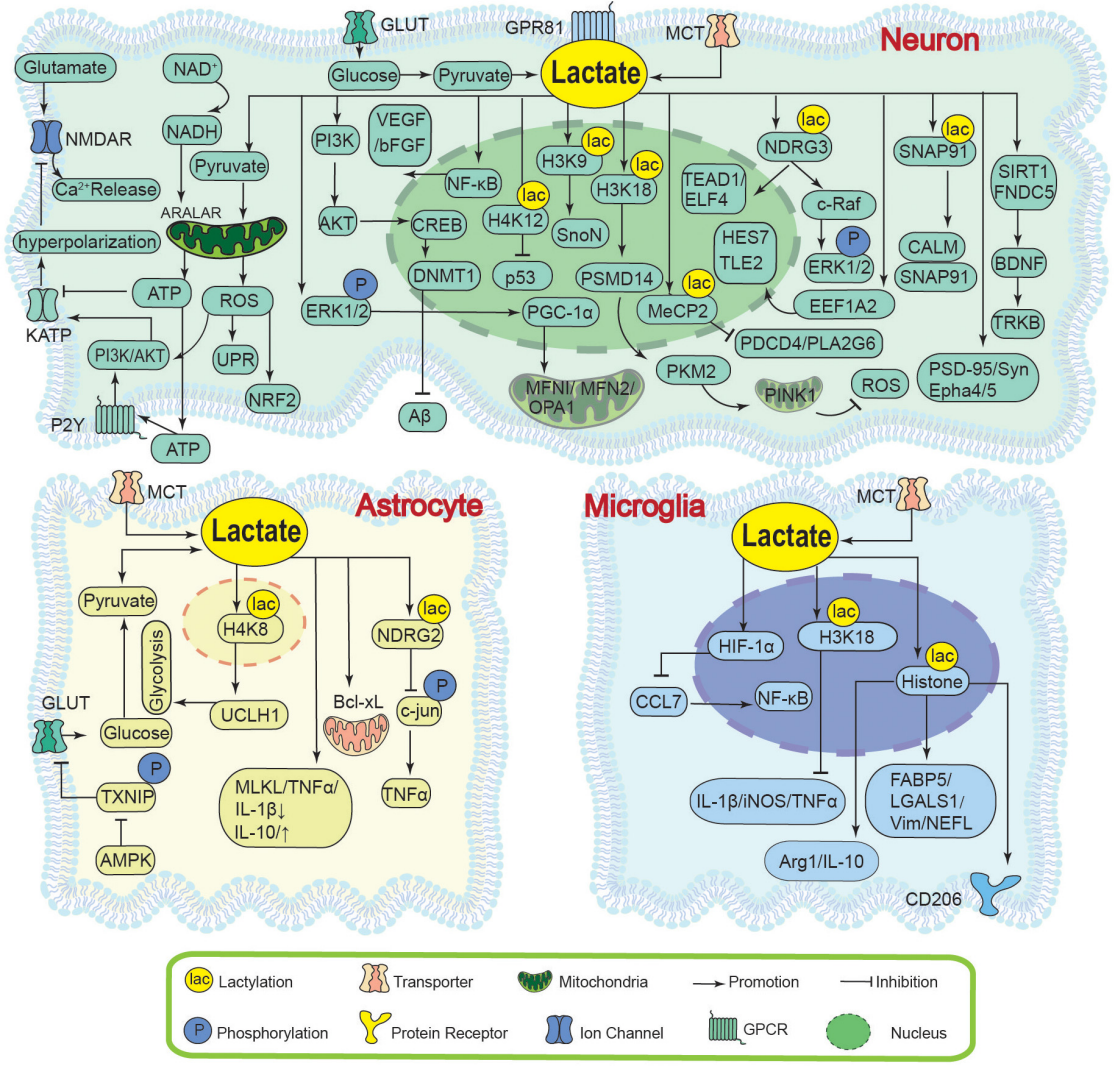
Neuroprotective effects of lactate. GLUT, Glucose Transporter; TXNIP, Thioredoxin-Interacting Protein; AMPK, AMP-activated protein kinase; NDRG2, N-myc Downstream-Regulated Gene 2; c-jun, c-jun; TNFα, Tumor Necrosis Factor Alpha; MCT, monocarboxylate transporter; HIF-1α, Hypoxia-Inducible Factor 1-Alpha; CCL7, C-C motif chemokine ligand 7; NF-κB, Nuclear Factor Kappa-B; H3K18, histone H3 lysine 18; VEGF, Vascular Endothelial Growth Factor; Arg1, Arginase 1; IL-6, Interleukin 6; Bcl-xL, B-cell lymphoma-extra Large; GPR81, G Protein-Coupled Receptor 81; NMDAR, N-Methyl-D-Aspartate Receptor; NAD^+^, nicotinamide adenine dinucleotide; NADH, reduced nicotinamide adenine dinucleotide; ERK1/2, Extracellular Signal-Regulated Kinases 1 and 2; ATP, adenosine triphosphate; KATP, ATP-sensitive potassium channel; P2Y, P2Y Purinergic Receptor; PI3K/AKT, Phosphatidylinositol 3-Kinase/Protein Kinase B; ROS, reactive oxygen species; NRF2, Nuclear Factor Erythroid 2-Related Factor 2; UPR, Unfolded Protein Response; CREB, cAMP Response Element-Binding Protein; p300, E1A-Binding Protein p300; DNMT1, DNA (Cytosine-5)-methyltransferase 1; Aβ, Amyloid-beta; PGC1α, Peroxisome Proliferator-Activated Receptor Gamma Coactivator 1-Alpha; TFAM, Mitochondrial Transcription Factor A; MFN1/MFN2/OPA1, Mitofusin 1/Mitofusin 2/Optic Atrophy 1; SIRT1, Sirtuin 1; FNDC5, Fibronectin Type III Domain-Containing Protein 5; BDNF, Brain-Derived Neurotrophic Factor; TRKB, Tropomyosin Receptor Kinase B; TEAD1/ELF4, TEA Domain Transcription Factor 1/E74-Like ETS Transcription Factor 4; VEGF/bFGF, Vascular Endothelial Growth Factor/Basic Fibroblast Growth Factor; EEF1A2/HES7/ELF4, Eukaryotic Elongation Factor 1 Alpha 2/Hairy and Enhancer of Split 7/E74-Like ETS Transcription Factor 4; PSD-95, Postsynaptic Density Protein 95; Syn, Synaptophysin; Epha4, Ephrin Type-A Receptor 4; PSMD14, Proteasome 26S Subunit, Non-ATPase 14; PINK1, PTEN Induced Kinase 1; PKM2, Pyruvate Kinase M2; SnoN, Ski-related novel protein N; SNAP91, Synaptosome-Associated Protein 91; CALM, Clathrin Assembly Lymphoid Myeloid Leukemia Protein; UCHL1, Ubiquitin C-terminal hydrolase L1; c-Raf, Raf-1; MeCP2, Methyl-CpG-Binding Protein 2; PDCD4, Programmed cell death 4; PLA2G6, Phospholipase A2 group VI.

Lactate primarily relies on the “lactate shuttle mechanism” to achieve metabolic crosstalk and signaling pathway activation among neural cells, thereby maintaining energy homeostasis and cellular structural integrity within the nervous system (Brooks, 2018; Chen et al., 2025; Figure 1).

Astrocytes serve as the primary lactate producers in the nervous system (Zhou et al., 2024). AMP-activated protein kinase (AMPK) enhances glycolysis and lactate accumulation by promoting the expression and membrane localization of Glucose Transporter 1 (GLUT1) through the phosphorylation-mediated degradation of Thioredoxin-Interacting Protein (TXNIP), thereby sustaining neuronal energy supply (Muraleedharan et al., 2020). After spinal cord injury, the Ubiquitin C-terminal Hydrolase L1 (UCHL1)/6-Phosphofructo-2-kinase/Fructose-2,6-bisphosphatase 3 (PFKFB3) axis is enhanced in astrocytes, leading to glycolytic hyperactivity and increased lactate production. The elevated lactate induces histone H4 lysine 8 lactylation (H4K8la), which in turn promotes transcription of UCHL1 and glycolysis-related genes, forming a glycolysis/H4K8la/UCHL1 positive feedback loop that supports neuronal energetics and inhibits ferroptosis (Xiong et al., 2025).

Neurons rely on the “lactate shuttle” mechanism to utilize astrocyte-derived lactate for energy metabolism and functional regulation (Dias et al., 2023). Lactate can be directly oxidized in the tricarboxylic acid (TCA) cycle and electron transport chain (ETC) or first converted to pyruvate to generate ATP, ultimately enhancing cerebral oxidative metabolism (Rabinowitz and Enerbäck, 2020; Vespa et al., 2025). Concurrently, ATP generated from lactate metabolism at a physiological concentration of 2–5 mM (elevating to 4–10 mM during neuronal activity) closes ATP-sensitive potassium channels (KATP), thus maintaining neuronal excitability and enhancing neuronal electrical conduction (Karagiannis et al., 2021; Yao et al., 2023). In addition, exercise-induced lactate promotes lactylation of the Synaptosome-Associated Protein 91 (SNAP91, synaptic protein) at residue K885, enhancing its interaction with Clathrin Assembly Lymphoid Myeloid Leukemia Protein (CALM) facilitating synaptic vesicle trafficking, which improves synaptic transmission efficiency and stress resilience (Yan L. et al., 2024).

In conclusion, lactate mediates metabolic crosstalk among neural cells via the “lactate shuttle” mechanism. Astrocytes enhance glycolysis to produce lactate through pathways such as AMPK and UCHL1/PFKFB3. Neurons utilize lactate for ATP generation via the TCA cycle and maintain excitability by closing KATP channels; lactate also induces lactylation of proteins like SNAP91 to regulate synaptic function. These mechanisms collectively sustain energy homeostasis and function of the nervous system.

### 2.2 Reduction of neuroexcitotoxicity and reactive oxygen species (ROS) levels

Lactate exerts an indispensable protective role in reducing neuronal excitotoxicity and oxidative stress through its metabolites and a series of signaling pathways activated thereby, with its effects showing significant concentration dependence (Figure 1).

Neuronal excitotoxicity is mainly because of the excessive activation of NMDA receptors and massive calcium ion influx induced by excessive glutamate release, and lactate can directly neutralize this toxicity (Verma et al., 2022; Nguyen et al., 2025). Lactate (5–10 mM, a physiologically relevant concentration in brain local microenvironment) is transported into neurons via MCTs and metabolized to pyruvate and NADH. Pyruvate-derived ATP is released extracellularly and activates the Phosphatidylinositol 3-Kinase (PI3K) signaling pathway via P2Y receptors, leading to KATP channel opening, neuronal hyperpolarization, and reduced calcium ions (Ca^2+^) influx through NMDA receptors, thereby preventing excitotoxic death (Jourdain et al., 2016). Concurrently, NADH supports mitochondrial function via the ARALAR-malate-aspartate shuttle (ARALAR-MAS), promoting neuronal survival when glucose utilization is impaired (Satrústegui et al., 2007; Yang et al., 2014; Llorente-Folch et al., 2016; Kanellopoulos et al., 2020). Beyond metabolism, lactate also modulates proteostasis and oxidative stress responses. Treatment with 20 mM lactate promotes histone H3 lysine 18 lactylation (H3K18la) and upregulates the expression of the Proteasome 26S Subunit, Non-ATPase 14 (PSMD14) gene. PSMD14 maintains the stability of Pyruvate Kinase M2 (PKM2) through deubiquitination, which in turn activates PTEN Induced Kinase 1 (PINK1)-mediated mitophagy, reduces ROS production, and inhibits neuronal PANoptosis (a combined form of pyroptosis, apoptosis, and necroptosis) (Xu L. et al., 2025).

In non-neuronal cells, lactate contributes to cytoprotection through regulation of autophagy and stress response pathways. Autophagy facilitates the clearance of damaged organelles and macromolecules, mitigates oxidative stress, and preserves mitochondrial function (Filomeni et al., 2015). Similarly, 10 mM lactate (in *Caenorhabditis elegans*) induces a mild ROS increase that activates protective pathways including the Unfolded Protein Response (UPR), Nuclear Factor E2-related Factor 2 (NRF2)-mediated antioxidant pathways, and the PI3K/Protein Kinase B (AKT) cell survival signaling pathway, enhancing proteostasis and extending lifespan. In contrast, 100 mM lactate intensifies oxidative stress but shortens lifespan (Tauffenberger et al., 2019; He et al., 2020).

To put it briefly, lactate prevents excitotoxic neuronal death by metabolically activating the PI3K pathway; promotes mitophagy via H3K18la-induced PKM2 stabilization; and activates cytoprotective pathways including UPR and NRF2-mediated antioxidant response through a mild induction of ROS. These integrated metabolic and epigenetic actions underlie lactate’s essential role in neuronal survival.

### 2.3 Alleviation of neuroinflammation

Lactate exerts a synergistic effect in alleviating neuroinflammation through multiple mechanisms, including activating specific signaling pathways, stabilizing fundamental proteins, and inducing epigenetic reprogramming, with its efficacy characterized by cell type specificity and concentration dependence (Figure 1).

In neurons, lactate exerts anti-inflammatory effects primarily through receptor-mediated signaling. Under ischemic and hypoxic conditions, 10 mM lactate (the optimal concentration, while 15 mM lactate showing attenuated effects) binds to the GPR81 receptors in cortical and hippocampal neurons, activating the PI3K/Akt signaling pathway (Zhang et al., 2021). This process leads to the phosphorylation of cAMP Response Element-Binding Protein (CREB) and subsequent expression of DNA methyltransferase 1 (DNMT1), thereby inhibiting Amyloid-beta (Aβ) deposition and neuroinflammation (Pugazhenthi et al., 2011; Amidfar et al., 2020).

In microglia, 10 mM lactate (*in vitro*) downregulates the expression of chemokine C-C motif chemokine ligand 7 (CCL7) by activating Hypoxia-Inducible Factor-1α (HIF-1α), thereby inhibiting the activation of the NF-κB signaling pathway and reducing the release of pro-inflammatory cytokines. However, the protective effect of 50 mM lactate is attenuated, which suggests that high concentrations of lactate may inhibit signal transduction and warrants further investigation (Zhang et al., 2025). Lactate can also directly alter the gene expression profile through lactylation, thereby inducing functional remodeling of immune cells (Zhang et al., 2019). Exercise or hypoxia-induced lactate promotes H3K18la, reducing pro-inflammatory gene (e.g., IL-1β, iNOS, TNF-α) expression, supporting neural repair and cognitive improvement (Han et al., 2023), while global protein lactylation alters the expression of Fatty Acid Binding Protein 5 (FABP5), Galectin 1 (LGALS1), Vimentin (Vim), and Neurofilament Light Chain (NEFL), inhibiting M1 and promoting M2 polarization through upregulation of anti-inflammatory mediators (e.g., Arg-1, CD206, IL-10) (Zhang et al., 2024).

In astrocytes, 30 mM lactate directly binds N-myc Downstream-Regulated Gene 2 (NDRG2) protein under oxygen-glucose deprivation, inhibiting its ubiquitination and suppressing c-Jun phosphorylation and Tumor Necrosis Factor-α (TNF-α), transcription, thereby blocking neuroinflammatory cascades (Xu et al., 2022). Moreover, 10 mM lactate (the effective concentration, *in vitro*) alleviates intracellular calcium overload, thereby mitigating neuroinflammation by downregulating expressions of pro-inflammatory genes including Mixed Lineage Kinase Domain-Like Protein (MLKL), TNF-α, and Interleukin-1β (IL-1β), and simultaneously upregulating transcription levels for anti-inflammatory genes including IL-10 as well as anti-apoptotic gene B-cell Lymphoma-extra Large (Bcl-xL) (Babenko et al., 2024).

In summary, in neurons, lactate suppresses neuroinflammation by binding to the GPR81 receptor and activating the PI3K/Akt pathway to upregulate DNMT1 expression; in microglia, it promotes a shift to the anti-inflammatory M2 phenotype by activating HIF-1α to inhibit the NF-κB pathway and by inducing histone H3K18 lactylation and global protein lactylation; and in astrocytes, it exerts anti-inflammatory effects by stabilizing NDRG2 protein to inhibit the c-Jun/TNF-α pathway, thereby downregulating pro-inflammatory factors and upregulating anti-inflammatory and anti-apoptotic genes.

### 2.4 Neuroplasticity and neurogenesis

Lactate plays a crucial role in enhancing synaptic plasticity and promoting neurogenesis and angiogenesis by regulating mitochondrial function, critical signaling pathways, and epigenetic modifications. Its mechanism is intricate, involving multiple cell types and molecular pathways (Figure 1).

In neurons, lactate can enhance the connection strength and function of the existing neuronal network, which serves as the fundamental basis for learning and memory (Shang et al., 2023). Upon entering neurons via MCT2, lactate promotes the expression of synaptic proteins (e.g., PSD-95, Syn), upregulates genes of the Eph receptor family (e.g., Epha4, Epha5), and facilitates axon guidance and synaptic plasticity (Han H. et al., 2025). Metabolically, lactate increases the intracellular NADH/NAD^+^ ratio, which enhances NMDA receptor activity and calcium influx, leading to the upregulation of neuroplasticity-related genes such as Activity-Regulated Cytoskeleton-Associated Protein (Arc), cellular Proto-Oncogene Fos (c-Fos), Zinc Finger Protein 268 (Zif268), and Brain-derived neurotrophic factor (BDNF), ultimately supporting memory function (Yang et al., 2014). Exercise-induced lactate elevation (12–20 mM) further strengthens synaptic plasticity through multiple signaling pathways. Lactate (approximately 12.3 ± 3.5 mM) binds to the GPR81 receptor on hippocampal neurons, which activates the Extracellular Signal-Regulated Kinase 1/2 (ERK1/2) signaling pathway. This activation enhances the activity the activity of Peroxisome Proliferator-Activated Receptor Gamma Coactivator 1 Alpha (PGC-1α), leading to the upregulation of mitochondrial biogenesis and fusion proteins (e.g., MFN1, MFN2, OPA1), and ultimately induces the expression of neuroplasticity-related genes to potentiate Long-Term Potentiation (LTP) (Shang et al., 2023). Alternatively, lactate (about 13–20 mM in blood) enters hippocampal neurons via blood circulation, activating sirtuin 1 (SIRT1), PGC-1α and fibronectin type III domain containing 5 (FNDC5) expression, and promoting BDNF release. BDNF subsequently activates downstream signaling pathways through the Tropomyosin Receptor Kinase B (TRKB) receptor, thereby enhancing learning and memory capabilities (El Hayek et al., 2019; Park et al., 2021). However, another study indicated that the elevated lactate level after exercise enters hippocampal neurons depending on MCT2 rather than lactate receptor HCAR1 (GPR81), which promotes adult hippocampal neurogenesis and increases the number of mature newborn neurons, but does not improve cognitive learning and memory function (Shi et al., 2024).

In neural stem and progenitor cells, lactate directly stimulates proliferation and differentiation to support neuroregeneration. Lactate directly stimulates the proliferation of neural progenitor cells (NPCs) by shortening the cell division cycle and increasing the proportion of dividing cells, thus providing a cellular foundation for neuroregeneration (Lev-Vachnish et al., 2019). In hypoxic conditions, lactate facilitates the differentiation of neural stem cells via both NDRG3-dependent and independent pathways. On one hand, lactate stabilizes NDRG3 by inhibiting its ubiquitination-mediated degradation (Wang et al., 2009; Yang et al., 2013). The resulting NDRG3-lactate complex then activates the cellular Raf-1 (c-Raf) and ERK1/2 pathway to drive cell growth and angiogenesis (Lee et al., 2015), while also translocating to the nucleus to promote neuronal differentiation by interacting with transcription factors TEA Domain Transcription Factor 1 (TEAD1) and E74 Like ETS Transcription Factor 4 (ELF4) (Xu et al., 2023). On the other hand, lactate directly targets Eukaryotic Translation Elongation Factor 1 Alpha 2 (EEF1A2), Hes Family BHLH Transcription Factor 7 (HES7) and Transtucin-Like Enhancer of Split 2 (TLE2) to achieve neurogenesis (Xu et al., 2023).

Following brain injury, lactate elevates the phosphorylation level of the Inhibitor of Nuclear Factor Kappa B Alpha (IκBα) and releases NF-κB into the nucleus, which subsequently activates the expression of Vascular Endothelial Growth Factor (VEGF) and fibroblast growth factor (bFGF), resulting in angiogenesis and neurogenesis (Zhou et al., 2018). Additionally, 20 mM lactate induces histone H3K9 lactylation (H3K9la), thereby activating Ski-related novel protein N (SnoN) transcription and regulating metabolic reprogramming to favor the differentiation of NSCs (Xu W. et al., 2025). Similarly, H4K12 lactylation upregulates the expression of pro-proliferative genes such as MDM2 and MDM4, and suppresses p53-mediated apoptosis, promoting hippocampal neurogenesis (Li Z. et al., 2025). Lactate also lactylates the non-histone protein Methyl-CpG Binding Protein 2 (MeCP2) at residues K210 and K249 via p300, consequently inhibiting transcription of apoptotic genes such as Programmed Cell Death 4 (PDCD4) and Phospholipase A2 Group VI (PLA2G6) and reducing neuronal death (Sun M. et al., 2025).

In conclusion, in neurons, lactate enhances synaptic plasticity and learning/memory by augmenting NMDA receptor activity to upregulate neuroplasticity-related genes, and by activating signaling pathways such as GPR81/ERK and SIRT1/PGC-1α/FNDC5. In neural stem cells, it promotes proliferation and differentiation by stabilizing NDRG3 to activate the Raf/ERK pathway or by inducing lactylation modifications of proteins including H3K9, H4K12, and MeCP2. Following brain injury, lactate further induces angiogenesis and neurogenesis via activation of the NF-κB pathway.

## 3 Neuroinjury roles of lactate and lactylation modification

Although lactate serves a vital neuroprotective function under physiological conditions, alterations in the body’s microenvironment, such as excessive lactate accumulation, contribute to nerve damage through pathways including energy metabolism disorders, mitochondrial dysfunction, oxidative stress, epigenetic modifications, and neuroinflammatory responses.

### 3.1 Abnormal lactate metabolism exacerbates nerve damage

**FIGURE 2:**
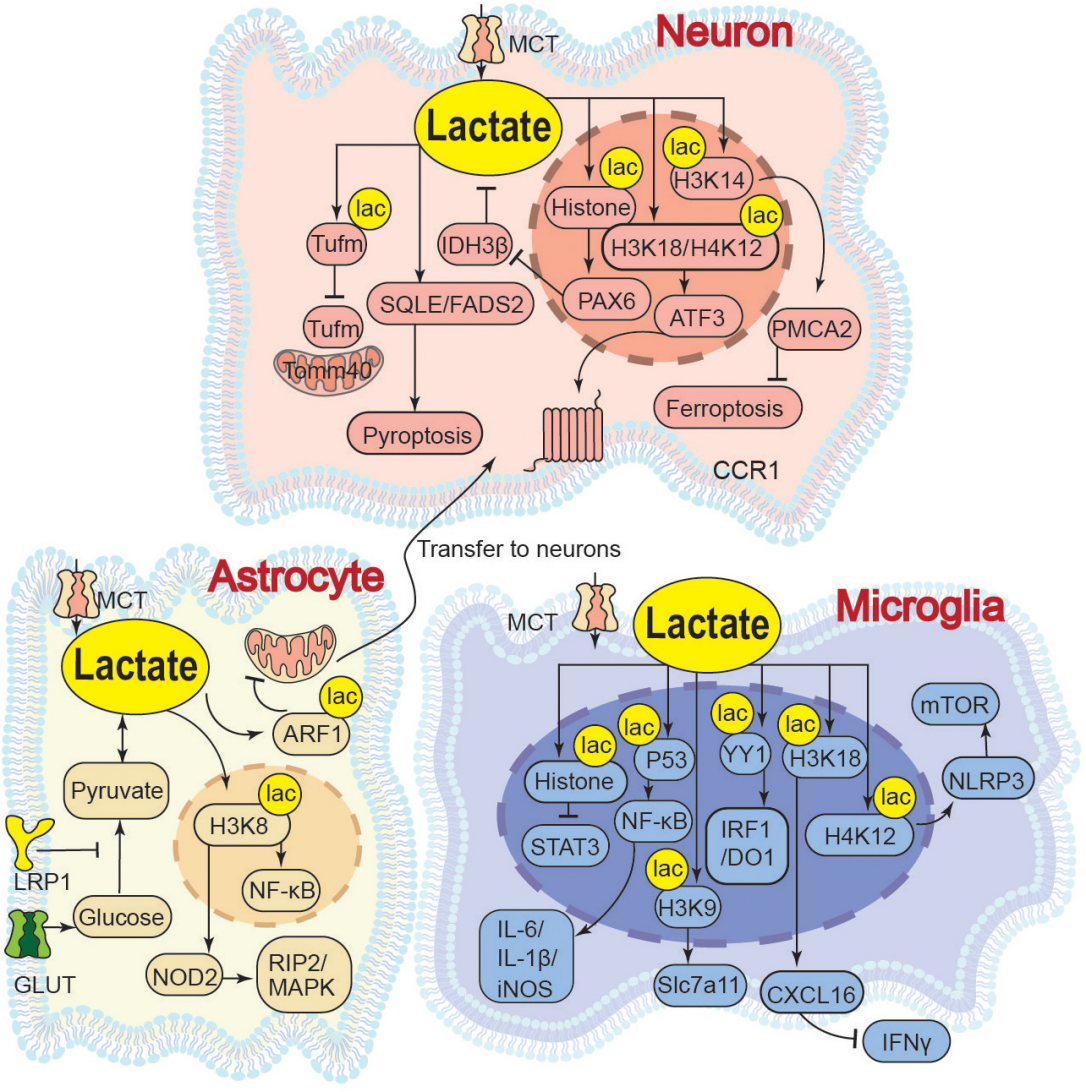
Neuroinjury effects of lactate. LRP1, Low-Density Lipoprotein Receptor-Related Protein 1; ARF1, ADP-Ribosylation Factor 1; YY1, Yin Yang 1; IRF1/DO1, Interferon Regulatory Factor 1/DNA Damage-Inducible 1; SEMA4D/CCL5/STAT3, Semaphorin 4D/C-C motif chemokine ligand 5/Signal Transducer and Activator of Transcription 3; Slc7a11, Solute Carrier; IL-1β, Interleukin 1 Beta; iNOS, Inducible Nitric Oxide Synthase; PEP, Phosphoenolpyruvate; PKM2, Pyruvate Kinase M2; P53, Tumor Protein P53; IκBα, Nuclear Factor Of Kappa Light Polypeptide Gene Enhancer In B-Cells Inhibitor Alpha; IDH3β, Isocitrate Dehydrogenase 3 Beta; PAX6, Paired Box 6; p-Tau, Phosphory tochondrial Translation Elongation Factor Tu; Tomm40, Translocase of Outer Mitochondrial Membrane 40; Acetyl-CoA, Acetyl Coenzyme A; PMCA2, Plasma Membrane Ca^2+^-ATPase 2; NOD2, Nucleotide-Binding Oligomerization Domain Containing 2; RIP2/MAPK, Receptor-Interacting Serine/Threonine-Protein Kinase 2/Mitogen-Activated Protein Kinase; STAT3, Activator of Transcription 3; NLRP3, NOD-like receptor family, pyrin domain-containing 3; mTOR, Mechanistic Target of Rapamycin; CXCL16, C-X-C Chemokine Ligand 16; IFNγ, Interferon Gamma; ATF3, Activating Transcription Factor 3; CCR1, C-C chemokine receptor 1; SQLE, Squalene Epoxidase; FADS2, Fatty Acid Desaturase 2.

In pathological conditions such as cerebral ischemia, traumatic brain injury, or aging, impaired metabolic pathways lead to the abnormal accumulation of lactate. High concentrations of lactate disrupt ionic homeostasis, induce oxidative stress, cause mitochondrial dysfunction, and exacerbate damage through aberrant lactylation, ultimately resulting in neuronal death and neurodegenerative diseases (Figure 2).

In neurons, lactate accumulation directly impairs neuronal function by acidifying the microenvironment and activating specific channels. At physiological levels, lactate activates Acid-Sensing Ion Channel 1a (ASIC1a) channels on neurons, enhancing mitochondrial respiration and reducing ROS production for metabolic coordination and neuroprotection (Yermolaieva et al., 2004; Lama et al., 2014; Azoulay et al., 2022). Conversely, a pathological “lactate storm” (>5 mM) induces extracellular acidosis, leading to ASIC1a overactivation, disrupted Ca^2+^ influx, and ultimately, neuronal apoptosis (Lama et al., 2014). This suggests that lactate at different concentrations activates ASIC1a channels to varying degrees, thereby mediating distinct biological effects. Additionally, intracellular lactate upregulates lipid metabolism genes such as Squalene Epoxidase (SQLE) and Fatty Acid Desaturase 2 (FADS2), resulting in abnormal aggregation of lipid droplets, which suppresses the expression of synaptic plasticity-related proteins and contributes to neuronal pyroptosis (Lan et al., 2024). Lactate accumulation disrupt the mitochondrial membrane potential, induce the opening of the mPTP, trigger the release of cytochrome C, and inhibit the activity of mitochondrial electron transport chain complexes, ultimately resulting in caspase-mediated apoptosis (Halestrap, 2009; Kalani et al., 2018; Cai et al., 2023). Pathological lactylation also contributes to neuronal injury. By inducing histone H3K14la to inhibit the Plasma Membrane Ca^2+^-ATPase 2 (PMCA2) expression, lactate causes intracellular Ca^2+^ overload, which promotes iron-dependent lipid peroxidation and ultimately exacerbates neuronal ferroptosis and brain injury (Sun T. et al., 2025). Similarly, pathological levels of lactate induce lactylation at the K286 site of the Mitochondrial Translation Elongation Factor Tu (Tufm). This non-histone lactylation modification impairs the interaction between Tufm and the Translocase of Outer Mitochondrial Membrane 40 (Tomm40), thereby blocking mitophagy and exacerbating neuronal apoptosis (Weng et al., 2025). In the peripheral nervous system (PNS), lactate exerts concentration-dependent dual functions on neuronal axons. Physiological levels support axonal energy metabolism and regeneration. However, chronic elevation to ∼10 mM (e.g., via Schwann cell Rheb knockout) causes lactate overload in axonal mitochondria. This initially boosts ATP production but ultimately induces excessive ROS, mitochondrial damage, and axonal degeneration (Jia et al., 2021).

In astrocytes, lactate concentration regulated by Low-density Lipoprotein Receptor-related Protein 1 (LRP1) determines the efficiency of mitochondrial transfer to neurons. Elevated lactate induces K73 lactylation on ADP-ribosylation factor 1 (ARF1), which directly impairs vesicle transport. This inhibition is concentration-dependent, with higher lactate levels leading to greater ARF1 lactylation and a stronger blockade of transfer (Zhou et al., 2024).

To conclude, in neurons, lactate induces calcium dyshomeostasis and apoptosis via ASIC1a overactivation, triggers abnormal lipid droplet accumulation and suppresses synaptic plasticity by upregulating SQLE/FADS2, and exacerbates ferroptosis and impairs mitophagy by inhibiting PMCA2 expression through H3K14 lactylation or by disrupting the Tufm-Tomm40 interaction through Tufm lactylation, respectively. In peripheral nerve axons, chronic lactate accumulation promotes mitochondrial ROS overproduction and axonal degeneration. In astrocytes, lactate inhibits mitochondrial transfer to neurons by inducing ARF1 lactylation.

### 3.2 Lactylation modification amplifies neuroinflammation

Elevated lactate concentrations and the resultant protein lactylation modifications play extensive roles in the pathogenesis of various diseases, including neurodegenerative disorders, neuropathic pain, and tumor immune evasion. These effects are primarily mediated through mechanisms such as disrupting cellular metabolic homeostasis, amplifying inflammatory responses, impairing DNA repair processes, and modulating immune cell functions (Pan et al., 2025; Figure 2).

In neurons, abnormal lactate metabolism drives neurodegenerative pathology through self-reinforcing feedback loops. In neurons of AD, increased lactate promotes histone lactylation, leading to Paired Box Protein 6 (PAX6) activation. This in turn suppresses isocitrate dehydrogenase 3β (IDH3β) activity, creating a IDH3β-lactate-PAX6-IDH3β positive feedback loop that exacerbates TCA cycle impairment and reduces ATP production (Wang X. et al., 2024). After peripheral nerve injury (PNI), sensory neurons upregulate the Amphiregulin-Epidermal Growth Factor Receptor (AREG-EGFR) pathway to activate PKM2-dependent glycolysis and lactate accumulation; lactate then mediates H3K18/H4K12 lactylation via p300, upregulating Activating Transcription Factor 3 (ATF3) and C-C Chemokine Receptor 1 (CCR1) to induce neuronal hyperexcitability and maintain neuropathic pain (Deng et al., 2025).

In microglia, lactate acts as a vital mediator of neuroinflammation, primarily exerting pro-inflammatory effects by promoting histone lactylation, which targets specific gene promoters to regulate transcription and activate downstream pathways. Injured-activated microglia and macrophages (IAMs) undergo glycolytic reprogramming to produce lactate, which induces histone H3K18 lactylation (H3K18la) and promotes C-X-C Chemokine Ligand 16 (CXCL16) gene transcription, and this CXCL16 in turn suppresses Interferon-gamma (IFNγ) production, thereby exacerbating neuronal death and spinal cord injury (Ge et al., 2025). Moreover, pathologically elevated lactate induces H4K12la binding to the NOD-like Receptor Pyrin Domain Containing 3 (NLRP3) promoter, activating the NLRP3/mTOR (Mechanistic Target of Rapamycin) pathway to inhibit autophagic flux and exacerbate microglial activation (Wang et al., 2025). Additionally, by promoting H3K9la to target the Solute Carrier Family 7 member 11 (Slc7a11) gene promoter, lactate facilitates the synthesis and release of pro-inflammatory precursors, thereby exacerbating dopaminergic neuron damage (Qin et al., 2025). Moreover, lactate enhances histone lactylation via the E1A Binding Protein P300/Chromobox Protein Homolog 3 (EP300/CBX3) complex, upregulates CD47 expression, inhibits Signal Transducer and Activator of Transcription 3 (STAT3) phosphorylation, suppresses the phagocytosis of microglia and macrophages, and thereby promotes immune escape (Wang S. et al., 2024). Beyond histone modification, lactate also mediates non-histone lactylation of transcription factors, directly enhancing their ability to regulate inflammation-related genes. Under hypoxic conditions, lactate (20 mM, *in vitro*) enhance p53 lactylation, driving NF-κB nuclear translocation and pro-inflammatory factor such as IL-6, IL-1β, and inducible Nitric Oxide Synthase (iNOS) transcription, synergistically amplifying metabolic stress and neuroinflammation (Fei et al., 2024). Moreover, P300 triggers the Lys183 lactylation of the transcription factor Yin Yang 1 (YY1) protein. This non-histone lactylation enhances YY1’s binding affinity to the promoters of inflammation-related genes including Interferon Regulatory Factor 1 (IRF1), Indoleamine 2,3-dioxygenase 1 (IDO1), and Semaphorin 4D (SEMA4D), thereby increasing inflammatory factor secretion and promoting microglial migration to amplify neuroinflammation (Huang et al., 2024). In addition to lactylation-dependent pathways, lactate also modulates microglial function through metaboloelectrical coupling mechanisms. In depression-associated AD, microglial glycolysis-driven lactate accumulation upregulates Kv1.3 channels (voltage-gated potassium channels), promoting Aβ-containing exosome release and cognitive decline. However, the activation of Kv1.3 is not achieved through direct lactylation, and the specific mechanism remains unclear (Liu et al., 2025).

In astrocytes, lactate modulates immunosuppressive effects through epigenetic reprogramming, thereby exacerbating neural damage. Lactate-induced H3K18la upregulates Nucleotide-Binding Oligomerization Domain Containing 2 (NOD2), activating Receptor-Interacting Serine/Threonine-Protein Kinase 2/Mitogen-Activated Protein Kinase (RIP2/MAPK) and NF-κB pathways and ultimately triggering pyroptosis (Li J. et al., 2025).

It is noteworthy that histone H3K18 lactylation (H3K18la) in microglia exhibits context-dependent and even opposing functional outcomes: under injury-induced conditions (this section), H3K18la promotes CXCL16 transcription and suppresses IFNγ, thereby exacerbating neuroinflammation (Ge et al., 2025); whereas under exercise-induced conditions (Section “2.3 Alleviation of neuroinflammation”), H3K18la suppresses pro-inflammatory genes such as IL-1β, iNOS, and TNF-α, thereby supporting neural repair (Han et al., 2023). We speculate that such divergence may stem from lactylation targeting different genomic loci, which subsequently activates distinct sets of genes, and from variations in lactate concentrations that influence the specificity of lactylation sites. However, neither of the cited studies specified the lactate levels involved, highlighting an important gap for future mechanistic clarification.

To summarize, in neurons, lactate exacerbates TCA cycle impairment by forming an IDH3β-lactate-PAX6-IDH3β positive feedback loop via histone lactylation, or maintains neuropathic pain by upregulating ATF3 and CCR1 through H3K18/H4K12 lactylation. In microglia, it aggravates neuroinflammation by promoting CXCL16 transcription to suppress IFNγ release via H3K18la, activating the NLRP3/mTOR pathway to inhibit autophagy via H4K12la, facilitating pro-inflammatory precursor release by targeting Slc7a11 via H3K9la, and enhancing NF-κB and inflammatory gene transcription through non-histone p53 and YY1 lactylation. In astrocytes, lactate induces pyroptosis by activating the RIP2/MAPK and NF-κB pathways via H3K18la.

## 4 Drugs and treatments

Drugs targeting lactate primarily modulate lactate metabolism and inhibit its signaling pathways. The more mature applications of these drugs are observed in the field of cancer therapy. These include agents that target lactate transporters, such as AZD3965 (Benyahia et al., 2021; Buyse et al., 2022), Syrosingopine (Zhang et al., 2014; Buyse et al., 2022) and AR-C155858 (Guan et al., 2019); that target LDH as Gallo flavin (Vettraino et al., 2013; Dunbar et al., 2014; Zhang et al., 2014; Guan et al., 2019; Buyse et al., 2022) and FX-11 (Le et al., 2010; Dunbar et al., 2014; Zhang et al., 2014); and other targeted drugs such as 2-deoxyglucose (2-DG) (Zhang et al., 2014) and Dichloroacetate (DCA) (Dunbar et al., 2014).

However, due to the complex dual regulatory mechanisms of lactate in the nervous system, there are still relatively limited drugs targeting lactate metabolism for the treatment of neurological diseases. Some drugs have already been employed to preclinical or clinical trials for neurological disorders such as epilepsy and mitochondrial encephalomyopathy (Table 1). The mechanisms underlying most existing drugs require further validation through neuropathological models. For instance, although DCA has shown potential benefits in managing epilepsy and mitochondrial encephalopathy, lactic acidosis, and stroke-like episodes (MELAS) syndrome by modulating pyruvate metabolism to decrease lactate production, its precise effects on neuroinflammation and synaptic plasticity warrant additional investigation (Kaufmann et al., 2006). Therefore, the development of precision drugs based on the lactate metabolic pathway and lactate modification represents an urgent field of nervous system disorders.

**TABLE 1:** Drugs for the treatment of neurological diseases by affecting lactate metabolism.

Drug name	Mechanism of action	Treatment of disease	References
Dichloroacetate (DCA)	Inhibits pyruvate dehydrogenase kinase (PDHK), activates the pyruvate dehydrogenase complex, promotes the conversion of lactate to pyruvate, and reduces lactate production.	Mitochondrial encephalomyopathies (such as MELAS syndrome), epilepsy	Lee et al., 2022; Wang P. et al., 2024
Metformin	Inhibits mitochondrial complex I, reduces hepatic gluconeogenesis, and indirectly regulates lactate metabolism; Activation of AMPK pathway improves energy homeostasis.	PD, AD	Ryu et al., 2020
α-lipoic acid	Enhances mitochondrial function, promotes lactate clearance, and improves oxidative stress-related disorders of lactate metabolism.	Diabetic peripheral neuropathy, stroke	Agathos et al., 2018
Ranolazine	Inhibits late sodium current (INa,L) in myocardium and neurons, reduces intracellular calcium overload, improves energy metabolism, and decreases lactate accumulation.	Ischemic stroke, neuropathic pain	Estacion et al., 2010; Aldasoro et al., 2016
Etomoxir	Inhibits carnitine palmitoyltransferase 1 (CPT1), blocks fatty acid β-oxidation, forces cells to use glucose for energy, and regulates lactate-pyruvate balance.	Amyotrophic lateral sclerosis (ALS), epilepsy	Trabjerg et al., 2020
GV-971	Regulates gut microbiota and reducing neuroinflammation	Mild to moderate AD	Yu et al., 2024
SR13800	Inhibits MCT1, leading to intracellular lactate accumulation and changes in the NADH/NAD^+^ ratio, affecting cell metabolism	Neuroblastoma	Khan et al., 2020
2-DG	Inhibits glycolysis, targeting of LDH, interference with astrocyte-neuron lactate shuttle (ANLS), reduction of ATP production, activation of potassium channel opening, and hyperpolarization of neuronal cell membranes	Epilepsy	Fei et al., 2020

## 5 Discussion and perspective

In brief, the traditional view of lactate as a mere “metabolic waste product” has been completely overturned. Instead, lactate acts as a metabolic hub and information carrier, exerting complex dual roles in the nervous system. The ultimate effect of lactate, whether beneficial or detrimental, is not determined by lactate alone but is highly dependent on a complex biological context, primarily comprising three dimensions: concentration, spatiotemporality, and target specificity.

On one hand, the effects of lactate are concentration-dependent. Low concentrations (∼10 mM) often serve as physiological signals (Jourdain et al., 2016; Karagiannis et al., 2021; Yao et al., 2023; Vespa et al., 2025), while high concentrations (>30 mM) tend to induce pathological effects (Jia et al., 2021; Dou et al., 2023; Weng et al., 2025). For example, in neural stem cells (NSCs), 20 mM lactate promote neuronal lineage differentiation via the H3K9la-SnoN axis-mediated metabolic reprogramming (Xu W. et al., 2025), whereas in mature neurons, lactate accumulation induce H3K14 lactylation, leading to calcium overload and subsequent neuronal damage (Sun T. et al., 2025). An essential unresolved question is: Does there exist a precise “threshold” that dynamically varies with cell type and pathological stage? Current studies are mostly descriptive of phenomena, and quantitative analysis and mechanistic investigation of this threshold should be prioritized in future research. While some studies indicate lactate signals through GPR81 to enhance synaptic plasticity (Shang et al., 2023), others show its entry via MCT2 promotes neurogenesis without improving memory (Shi et al., 2024). And we speculate that it may stem from differential affinity of lactate for these receptors or transporters at varying concentrations.

On the other hand, the same lactate exerts drastically different effects at different times and locations. For example, the very same modification, H4K12la, can drive starkly opposing biological outcomes depending on the cell type. In microglia, H4K12la exacerbates microglial activation and neuroinflammation by activating the NLRP3/mTOR pathway and suppressing autophagic flux (Wang et al., 2025). In neurons, however, H4K12la promotes adult hippocampal neurogenesis by inhibiting the pro-apoptotic and cell cycle arrest functions of p53 (Li Z. et al., 2025). Transient elevation of lactate after exercise acts as an adaptive signal, inducing beneficial synaptic plasticity and neurogenesis (Han H. et al., 2025; Xu W. et al., 2025). In contrast, sustained lactate accumulation in chronic diseases functions as a damaging signal, leading to irreversible epigenetic reprogramming (Dou et al., 2023; Liu et al., 2025; Weng et al., 2025). Furthermore, lactate homeostasis maintained intracellularly via specific mechanisms often confers neuroprotective effects (Babenko et al., 2024; Han H. et al., 2025), whereas extracellular abnormal accumulation causing acidosis typically activates specific signaling pathways to amplify vicious cycles (Wang X. et al., 2024; Hong et al., 2025). Lactate oxidation for energy production in neurons is protective (Brooks, 2018; Chen et al., 2025), while its induction of excessive reactive oxygen species (ROS) generation in mitochondria is destructive (Azoulay et al., 2022).

Finally, the discovery of lactylation modification has elevated lactate’s role to the level of precise regulation. Lactylation is a post-translational modification process wherein lactate serves as the substrate for the covalent modification of lysine residues on proteins. Current evidence highlights lactylation as a pivotal modification in the nervous system, targeting histone residues (H3K9, H3K18, H4K8, H4K12) and non-histone proteins (MeCP2, SNAP91, ARF1), thereby mediating diverse epigenetic and signaling events. However, two vital questions remain unresolved: first, whether there exists a pivotal lactate concentration threshold that triggers the modification of specific lysine residues on histones and non-histone proteins, second, whether the specific lactylation of certain lysine residues in target proteins is determined by the specificity of enzymatic reactions (e.g., AARS1/2, p300) or by protein structure and local microenvironment. These issues warrant further exploration to provide a new theoretical basis for the treatment of nervous system diseases through the precise regulation of lactylation modification.
